# Gut instinct: microbiome as a modifiable target in the management of neurologic symptoms in myoclonic epilepsy with ragged-red fibers (MERRF)

**DOI:** 10.1097/MS9.0000000000003777

**Published:** 2025-08-28

**Authors:** Amna Rahman, Muneeb Faiz, Maliha Khalid, Muhammad Talha, Aminath Waafira

**Affiliations:** a Faisalabad Medical University/Punjab Medical College, Faisalabad, Pakistan; b King Edward Medical University, Lahore, Pakistan; c Jinnah Sindh Medical University, Karachi, Pakistan; d School of Medicine, The Maldives National University, Malé, Maldives

**Keywords:** gut microbiome, MERRF syndrome, mitochondrial disease, neurologic symptoms

## Abstract

Myoclonic epilepsy with ragged-red fibers (MERRF) is a rare mitochondrial disorder primarily driven by mutations in mitochondrial DNA, particularly the m.8344A>G variant in MT-TK, and is characterized by epilepsy, myoclonus, ataxia, and other multisystemic features. With no curative therapy, recent attention has turned to the gut microbiome as a modifiable factor influencing neurologic symptoms in mitochondrial diseases. Dysbiosis—induced by antibiotics, diet, or preservatives—has been linked to altered microbial metabolites such as short-chain fatty acids and indoxyl sulfate, which may exacerbate neurological dysfunction. Preliminary clinical trials and preclinical studies suggest that probiotics and dietary interventions can modestly improve disease burden and symptoms such as constipation. However, significant challenges remain, including lack of standardization in analytical protocols, heterogeneous host-microbiota responses, and inadequate patient stratification. To fully realize the therapeutic potential of microbiome-based approaches in MERRF, coordinated multicenter trials, clear regulatory guidelines, and machine learning-enhanced stratification will be essential.

*To the Editor,*

The genetic diseases caused by mutations in the genes of mitochondrial DNA (mtDNA) or nuclear DNA are referred to as primary mitochondrial disorders (MIDs). Myoclonic epilepsy with ragged-red fibers (MERRF) syndrome is one of the most common syndromic MIDs. It’s a rare syndromic, multisystem MID whose treatment is challenging, and only symptomatic measures can be taken against it. Classic MERRF, MERRF plus, or MERRF overlap syndromes are its phenotypical manifestations. Their symptoms may include generalized epilepsy, myoclonus, ataxia, migraine, intellectual disability, etc. Its prevalence is estimated to be less than 1 in 100 000. The pathogenic variant m.8344A>G in MT-TK is responsible for approximately 80% of cases of this condition. Epilepsy is one of the most common features of classic MERRF and MERRF plus^[1]^.

In recent studies, there is found an association between mitochondrial diseases such as MERRF and alterations in the gut microbiome has been found. Dysbiosis is caused by antibiotic use, food preservatives, and diet. It can lead to immune system dysfunction, metabolic disorders, and various neurologic diseases^[2]^. Bacterial metabolites such as short-chain fatty acids, secondary bile acids, and indoxyl sulphate are recognized as significant contributors to neurologic symptoms. So, therapeutic targets are these metabolites, which are also an exciting topic of research. All this suggests that dealing with dysbiosis can lead to reducing neurological symptoms in MERRF syndrome^[3]^.

Given its importance, an early-phase clinical trial and preclinical studies have been initiated to evaluate probiotic and symbiotic interventions for the restoration of healthy microbiome profiles and mitochondrial dysfunction. For instance, randomized controlled trials conducted in populations suffering from neurodegenerative disorders demonstrated that microbiome interventions, including probiotics or dietary changes, produce statistically significant, although modest, improvements in overall disease burden (standard mean difference, −0.57; 95% CI −0.93 to −0.21; *P* = 0.002), as well as improvements in constipation that is an often troubled complaint in mitochondrial syndromes. The interventions also induce changes in gut microbiome composition, although some responses can be heterogeneous, and the mechanisms are not fully elucidated^[4]^.

Major challenges obstruct the clinical acceptance of microbiome therapies for MERRF. The major roadblocks include a lack of standardization in the analytical protocols used to profile and validate microbiome-based therapies, and the few definitions that are in common usage. Above all, there is an urgent need to harmonize efforts aimed at regulation and methodology in order to promote larger and improved clinical studies, and to validate biomarkers from the microbiome. The absence of longitudinal studies, variability in host-microbiota interactions, and poor patient stratification tactics only further complicate against therapeutic standardization and integration into mainstream neurometabolic care^[5,6]^.

Recent research increasingly highlights the gut microbiome as both a contributing factor to neurological symptoms in MERRF syndrome and a potential therapeutic target. To advance this field, there is a pressing need for standardized analytical methods, clear regulatory frameworks, and the use of machine learning for patient stratification. Achieving meaningful outcomes will require coordinated multicenter trials and international collaboration to effectively translate microbiome-based therapies into real-world benefits for those affected by MERRF. Our work is in line with the TITAN Guidelines on the need for transparency in AI use in healthcare^[7]^.

## Data Availability

No data were generated for this manuscript.

## Peer and provenance statement

This article was not commissioned and was externally peer-reviewed.
